# Selective Targeting to Glioma with Nucleic Acid Aptamers

**DOI:** 10.1371/journal.pone.0134957

**Published:** 2015-08-07

**Authors:** Shraddha Aptekar, Mohit Arora, Clare Louise Lawrence, Robert William Lea, Katherine Ashton, Tim Dawson, Jane Elizabeth Alder, Lisa Shaw

**Affiliations:** 1 School of Pharmacy and Biomedical Sciences, College of Clinical and Biomedical Sciences, University of Central Lancashire, Preston, Lancashire, PR1 2HE, United Kingdom; 2 Department of Neuropathology, Lancashire Teaching Hospitals (NHS trust), Preston, PR2 9HT, United Kingdom; Consiglio Nazionale delle Ricerche (CNR), ITALY

## Abstract

Malignant glioma is characterised by a rapid growth rate and high capacity for invasive infiltration to surrounding brain tissue; hence, diagnosis and treatment is difficult and patient survival is poor. Aptamers contribute a promising and unique technology for the *in vitro* imaging of live cells and tissues, with a potentially bright future in clinical diagnostics and therapeutics for malignant glioma. The binding selectivity, uptake capacity and binding target of two DNA aptamers, SA43 and SA44, were investigated in glioma cells and patient tissues. The binding assay showed that SA43 and SA44 bound with strong affinity (K_d_, 21.56 ± 4.60 nM and K_d_, 21.11 ± 3.30 nM respectively) to the target U87MG cells. Quantitative analysis by flow cytometry showed that the aptamers were able to actively internalise in U87MG and 1321N1 glioma cells compared to the non-cancerous and non-glioma cell types. Confocal microscopy confirmed staining in the cytoplasm, and co-localisation studies with endoplasmic reticulum, Golgi apparatus and lysosomal markers suggested internalisation and compartmentalisation within the endomembrane system. Both aptamers selectively bound to Ku 70 and Ku 80 DNA repair proteins as determined by aptoprecipitation (AP) followed by mass spectrometry analysis and confirmation by Western blot. In addition, aptohistochemical (AHC) staining on paraffin embedded, formalin fixed patient tissues revealed that the binding selectivity was significantly higher for SA43 aptamer in glioma tissues (grade I, II, III and IV) compared to the non-cancerous tissues, whereas SA44 did not show selectivity towards glioma tissues. The results indicate that SA43 aptamer can differentiate between glioma and non-cancerous cells and tissues and therefore, shows promise for histological diagnosis of glioma.

## Introduction

The term glioma encompasses all tumours of glial cell origin, and is the most frequent brain tumour observed [1–3]. According to the world health organisation (WHO) classification, gliomas are categorised according to the grade, cell type, and location of the tumour. These include astrocytic tumours, namely, WHO classification grades I and II (astrocytoma), III (anaplastic astrocytoma) and IV (glioblastoma), oligodendrogliomas, ependymomas and mixed gliomas [4]. Despite recent advances in understanding the molecular heterogeneity of the disease and development of multimodal therapy, customised therapy for the most malignant and lethal form, glioblastoma (GB), remains challenging [5,6,7]. Such intrinsic heterogeneity in human glioma has meant there is a need for targeting ligands that can aid in the identification of tumour specific signatures.

Aptamers are highly specific molecular ligands used for targeting cell surface or internalised molecules that are expressed differentially in tumour cells and tissues [8–10]. Aptamers are composed of short oligonucleotides with etymology stemming from the Greek word “aptus” meaning “to fit” [11–13]. The development of artificial RNA (now known as aptamer) and Systemic Evolution of Ligands by Exponential enrichment (SELEX) process in 1990 by three independent groups namely Sullenger *et al*. [14], Tuerk and Gold [11], and Ellington and Szostak [12] significantly accelerated the generation of aptamers with a capacity of recognizing virtually any class of target molecules with high affinity and specificity. Since then several SELEX methods have been designed to generate aptamers against various targets conveniently and easily [15–17]. Amongst, various targets, aptamers generated by cell-SELEX offers an exceptional ability to discover unknown biomarkers associated or overexpressed in a specific cell type [18]. The nucleotide sequence in an oligonucleotide chain allows internal Watson and Crick base pairing (guanine to cytosine, adenine to thymine/ uracil) within the single stranded molecule. The folding pattern of such base pairing is known as the secondary structure of the aptamer [19]. Aptamers are known to bind to target molecules on single stranded regions known as a ‘bulge’ or ‘loop’, which contribute to the spatial structure of binding [20–22]. The formation of unique three—dimensional structure by aptamers is mainly due to changing intramolecular base pairing, having a combination of stems, loops, quadraplexes, pseudoknots, bulges, and hairpins. Such highly defined three-dimensional structures helps the aptamers to bind to the target molecules in a similar manner to conventional antibodies. Aptamers, however, can provide an excellent alternative to antibodies as they are non-immunogenic, have high affinity and sensitivity, low detection limits, and no batch-to-batch variation [23–25]. Such unique properties enable aptamers to be of value for diagnostics [26], ligand purification [27], target validation [28], biomarker discovery [29], and therapeutic interventions [26,30].

The use of aptamers in studying glioma is currently evolving. Researchers have reported a differential cell-SELEX technique using glioma cells, yielding aptamers that can selectively bind to the various human glioma cell lines [31–33]. The GMT8 aptamer was shown to have highest binding affinity for U87MG cells compared to cell lines of other origin including MCF-7, CEM, RAMOS, HT29 and HBE-135 [31]. Cerchia *et al*., (2009) reported a differential cell-SELEX using target U87MG cells, yielding aptamers that selectively bind to the tumorigenic glioma cell lines [32]. The nuclease-resistant RNA aptamers were developed through the cell-SELEX process and were capable of binding at high affinity to the target U87MG cells compared to the non-tumorigenic T98G cells. Likewise, aptamers such as GBM128 and GBM131 have been developed to target glioma cells [33]. While aptamers show great promise in selective targeting of glioma cell lines compared to non-cancerous cells, this is not enough because glioma is a heterogeneous disease. The study therefore, needed to be extended to glioma tissues as clinical tissue sections are more representative of the diseased state. The combined selectivity of aptamers for both live glioma cells and fixed glioma tissues from various patients with different grades would be more effective, as aptamers could potentially assist clinical diagnosis of glioma. From the literature, it was identified that there was a gap in the potential use of the aptamers for glioma diagnosis. To date, there is only one report on the use of aptamers for selectively recognising glioma tissues [33]. Although the report provided excellent data on the selectivity of aptamer GBM128 and GBM131 to glioblastoma tissues compared to the normal astroglial tissue and other human cancer tissues, the sample number was limited and therefore, conclusions were based on observing only one sample tissue without performing any statistical analysis which may produce false positive results. Moreover, the study did not differentiate between different grades of glioma. The aim of the current study presented here was to analyse novel DNA aptamer binding in a larger number of patient tissue samples and various cell lines and, most importantly, to determine the target for aptamer binding. Two out of eight RNA aptamers from Cerchia *et al*., (2009) (GL43 and GL44) were selected for the study as they had the lowest K_d_ (dissociation constant) and thus higher binding affinity towards U87MG cells [32]. These aptamers were generated through the standard cell SELEX process with selection and counter selection against U87MG (target) and T98G cells respectively at 37°C. The DNA homologs of the random sequences from these two published RNA aptamers were evaluated for their binding affinity, cellular localisation, uptake process, effect on cell viability and target identification on live glioma, non-cancerous and non-glioma cell lines.

## Materials and Methods

Human glioma cell lines 1321N1 (malignant astrocytoma, sub-clone of grade IV U118MG, tumorigenic *in vivo*), U87MG (grade IV glioblastoma, tumorigenic *in vivo*), and non-cancerous foetal astrocytes SVGp12 were obtained from the European Collection of Cell Cultures (ECACC, England, UK) and American Type Culture Collection (ATCC, Middlesex, UK). Two non-glioma cell lines, MCF-7 (breast cancer) and T24 (bladder cancer) were kindly donated by Royal Preston Hospital, UK and University of Leeds, UK respectively with their original commercial source from ECACC, England, UK. All media and supplements including Dulbecco’s modified eagle medium (DMEM), Eagle’s minimum essential medium (EMEM), foetal bovine serum (FBS), L-glutamine, Sodium pyruvate solution, non-essential amino acid (NEAA) and penicillin/streptomycin (pen/strep) were obtained from Scientific Laboratory Supplies (SLS), Lonza, Nottingham, UK. All other chemicals were purchased from Sigma-Aldrich Ltd (Butterworth, UK) unless otherwise stated.

### Cell Culture

1321N1 and T24 cell lines were grown in complete DMEM supplemented with 10% FBS and 2 mM L-glutamine whereas, U87MG, SVGp12 and MCF-7 cell lines were grown in complete EMEM supplemented with 10% FBS, 2 mM L-glutamine, sodium pyruvate (5 mM) and non-essential amino acids (5 mM). All cell lines were routinely grown in 75 cm^2^ or 25 cm^2^ tissue culture flasks and were maintained in a 37°C humidified incubator supplied with 5% CO_2_. Cell lines were harvested when they reached 70–80% confluence and were used between passages 5–25.

### Oligonucleotide synthesis and labelling

Aptamers GL43 and GL44 RNA were adapted from Cerchia *et al*., (2009) and modified by truncating the primer sequences from both ends. The DNA homologs of the shortened aptamers were synthesised by Integrated DNA technologies (IDT) and conjugated at the 5’ end with either Cy3 or with biotin and thereafter were named SA43 and SA44 (Table 1) (IDT, Glasgow, UK). For all binding experiments, random nucleotide sequences (random aptamer) with same length and nucleotide composition as the shortened aptamers were used as negative controls.

**Table 1:** Primary sequences of aptamers used in the study.

Aptamer	Sequence 5’ to 3’
SA43 DNA	ACG TTA CTC TTG CAA CAC AAA CTT TAA TAG CCT CTT ATA GTT C
SA44 DNA	ACG TTA CTC TTG CAA CAC CCA AAC TTT AAT AGC CTC TTA TAG TTC

### Determination of binding affinity of DNA aptamers to cell lines by flow cytometry

U87MG cells were seeded in 12-well plates at a density of 40000 cells/ well in complete media and were allowed to attach and grow for 36 hours at 37°C in a 5% CO_2_ humidified incubator. The binding affinity of Cy3 labelled aptamers SA43 and SA44 was determined by incubating varying concentrations (0.5 nM– 200 nM) diluted in media with live adherent U87MG cells for 90 mins at 37°C in a 5% CO_2_ humidified incubator. Untreated cells were incubated with complete growth medium alone. Post incubation, the cells were washed three times with 0.1 M PBS (pH 7.4) and detached using 0.25% trypsin EDTA for 2 minutes, centrifuged at 224 x g for 5 minutes and the supernatant that contained unbound aptamer was retained. Cells were resuspended in PBS for flow cytometry analysis. Fluorescence analysis was performed on a BD FACSAria flow cytometer (BD Biosciences, San Jose, CA, USA) using phycoerythrin (PE) (excitation at 488 nm and emission at 578 nm) and 10,000 events were collected for each sample. The mean fluorescent intensity (MFI) was determined using FACS Diva version 4.1.2 software (BD Biosciences). For calculation of the equilibrium dissociation constant (K_d_) of the aptamers and U87MG cell interaction, the average MFI value from at least three independent experimental repeats was plotted against aptamer concentration and a curve fitted using non-linear regression analysis (Graphpad Prism, Graphpad software, Inc, La Jolla, USA). Accordingly, the K_d_ value was obtained for each aptamer using the one site-specific binding equation (Eq 1) [34].

$$Aptamer_{bound}\;=\;\left(B_{max}\;\times \;C_{aptamer}\right)÷\left(K_{d}\;+\;C_{aptamer}\right)$$ (1)

Where: B_max_ = maximum binding sites; C_aptamer_ = concentration of aptamer; K_d_ = dissociation constant (binding affinity).

### Cellular uptake and localisation of aptamers by confocal microscopy

Cy3 labelled DNA aptamers were incubated with live glioma (U87MG, 1321N1), non-glioma (SVGp12) and other malignant cell lines (MCF7 and T24) and confocal imaging was used to obtain z-stacks to determine whether aptamer uptake or binding was selective and where the aptamer localised.

To assess the uptake of the aptamers in live glial and non-glial cells, glioma cells (U87MG and 1321N1), non-cancerous glial cells (SVGp12), breast cancer cells (MCF-7) and bladder cancer cells (T24) were plated on coverslips (round 12 mm, Harvard apparatus, Kent, UK) in 24 well plates at a seeding density of 30000 cells/ well in media and allowed to grow for 36 hours. Post attachment, the cells were incubated with 100 nM Cy3 labelled SA43, SA44 or random sequence aptamer for 90 minutes. Untreated cells were incubated with complete growth medium alone. Post incubation, the cells were washed three times with 0.1 M PBS, pH 7.4 to remove any unbound aptamer. Cells were fixed with 4% paraformaldehyde (PFA) for 15 minutes at room temperature. After fixing, the cells were washed three times with 0.1 M PBS, pH 7.4 and counter-stained using Vectashield mounting medium with DAPI (1.5 μg/ml) (Vector laboratories, Peterborough, UK). The cells were then visualised under 40x magnification using a Zeiss LSM510 confocal microscope (Zeiss LSM, Germany) (Excitation: 543 nm; emission: 560–615 nm). The images of aptamer uptake to the cells were acquired under 40x oil immersion objective in a Z- stack mode with independent slice thickness determined by the software. For all the staining experiments, cellular localisation was further confirmed by overlaying the fluorescence images (single plane image from the middle of Z-stack) with the nuclear DAPI stain.

### Cellular uptake and internalisation of aptamers by flow cytometry

Cell lines were prepared in 12-well plates prior to flow cytometry as described above. Post attachment, the cells were incubated with 100 nM Cy3 labelled aptamers SA43, SA44 or random aptamer (RA) for 90 minutes. Untreated cells were incubated with complete growth medium alone. To determine the binding selectivity and affinity for each aptamer on all cell lines, statistical analysis was performed on the mean fluorescent intensity (MFI) values using IBM SPSS statistics v20 software (IBM SPSS Statistics for Windows, Version 20.0, Armonk, NY). MFI values of the untreated cells were subtracted from the MFI values obtained from aptamer treated cells. Thereafter, the MFI of the random aptamer was subtracted from the MFI of the lead aptamers. A Shapiro-Wilk’s test was performed on the average MFI values from three individual experiments of each aptamer to test the normal distribution of the data. Statistical significance of differences in the means of average MFI values of each shortened aptamer between individual cell groups was then determined by using a one-way ANOVA followed by post hoc Bonferroni test [35]. The significant statistical difference was defined according to a *p* value less than 0.05.

### Effect of temperature on aptamer binding to the cells

Cells (U87MG, 1321N1 and SVGp12) were seeded into two 12-well plates and incubated with each aptamer (100 nM) at 4°C and 37°C simultaneously for 90 minutes. After incubation, cells were prepared following the aforementioned protocol for flow cytometry analysis. The binding assay experiments were repeated at least three times and were analysed using WinMDI 2.9 software. Statistical significance of differences in the means of average MFI values of each DNA aptamer between individual cell groups treated at 4°C and 37°C was then determined by using two-way ANOVA followed by Bonferroni post-hoc test [35].

### Determining subcellular localisation of the aptamers SA43 and SA44 DNA

To study the subcellular localisation of aptamers, U87MG cells were plated on coverslips on 24-well plates at a seeding density of 20000 cells/well in media and allowed to grow for 24 hours. Post attachment, live cells were treated with 100 nM of SA43, SA44 and RA for 24 hours to reveal the subcellular structures. On the same day, cells were transfected with CellLight Golgi-GFP, BacMan 2.0 and CellLight ER- GFP, BacMan 2.0 (ThermoFisher Scientific, Leicestershire, UK) according to the manufacturer’s instructions and incubated overnight at 37°C in a 5% CO_2_ humidified incubator to track co-localisation of aptamers with golgi apparatus and endoplasmic reticulum respectively. Lysotracker green DND-26 (100 nM) (ThermoFisher Scientific, Leicestershire, UK) was added to the cells and incubated for 2 hours to track lysosomal co-localisation. Post incubation, the cells were washed three times with 0.1 M PBS, pH 7.4 to remove any unbound aptamer and marker. Cells were fixed with 4% PFA for 15 minutes at room temperature. After fixing, the cells were washed three times with 0.1 M PBS, pH 7.4 and counter-stained using Vectashield mounting medium with DAPI (1.5 μg/ml) (Vector laboratories, Peterborough, UK). The cells were then visualised and acquired under 63x magnification using a Zeiss LSM510 confocal microscope (Zeiss LSM, Germany) (Cy3 channel-excitation: 543 nm/emission: 560–615 nm; GFP channel- excitation: 488 nm/emission: band pass filter 505–530). The co-localisation analysis was performed using Zeiss LSM image browser v4 and Image J v1.47 software.

### Effect of aptamers on cell viability

The effect of aptamers on cell viability was tested using the PrestoBlue assay (Sigma-Aldrich Ltd, Butterworth, UK). Briefly, 2000 cells/ well of U87MG, 1321N1 and SVGp12 cells were seeded in 96-well plates and incubated for 24 h. Cells were treated with four different concentrations (20 nM, 100 nM, 500 nM and 1000 nM) of biotinylated DNA aptamers SA43 or SA44 for 24, 48 and 72 hours. Cisplatin diluted in media (10 μM) was used as a positive control. To evaluate the cell viability following 24, 48 and 72 hours treatment, 10 μl of PrestoBlue was added to each well and incubated for 1 hour at 37°C. The assay was read using a TECAN genios pro multifunctional microplate reader (Tecan, Austria; software version 4.53) with excitation 535 nm and emission 612 nm. Cell viability was expressed in percentage relative to control untreated cells grown in media.

### Protein extraction from cultured cell lines and aptamer precipitation

Native protein extracts were prepared from 70–80% confluent U87MG or 1321N1 cells. Trypsinised cells were washed in cold 0.1 M PBS, pH 7.4 then lysed in 0.1% Triton X-100 in PBS. Following lysis, the extract was centrifuged at 4472 x g at 4°C for 10 minutes and the protein concentration determined using the Bradford assay.

The protein extract at 2.5 mg/ ml was incubated with 100 nM of the biotin labelled aptamer for 45 minutes at 4°C. 100 μl of streptavidin agarose beads (ThermoFisher Scientific, Leicestershire, UK) were washed in 0.1 M PBS, pH 7.4, before being added to the sample and incubated for 30 minutes at 4°C. The streptavidin agarose was washed three times in 0.1 M PBS, pH 7.4 before bound proteins were eluted by incubating at 95°C for 5 minutes in SDS sample buffer (sodium dodecyl sulphate 10%, glycerol 50%, dithiothreitol 0.5 M, bromophenol blue 0.25%, tris-Cl 0.25 M, pH 6.8). 15 μl of the sample was run on 10% SDS PAGE resolving gels with 4% stacking gel and stained with Coomassie blue.

### Mass spectrometry analysis

Protein bands were excised from the gel and analysed by nLC-ESI-MS/MS at St. Andrews University, Dundee. Protein samples were concentrated using a SpeedVac (ThermoSavant) and then separated on an Acclaim PepMap 100 C18 trap and an Acclaim PepMap RSLC C18 column (ThermoFisher Scientific) using a nanoLC Ultra 2D plus loading pump and nanoLC as-2 autosampler (Eskigent). The peptides were eluted with a gradient of increasing acetonitrile, containing 0.1% formic acid (5–40% acetonitrile in 5 min, 40–95% in a further 1 min, followed by 95% acetonitrile to clean the column, before re-equilibration to 5% acetonitrile). The eluent was sprayed into a TripleTOF 5600 electrospray tandem mass spectrometer (ABSciex) and analysed in Information Dependent Acquisition (IDA) mode, performing 250 msec of MS followed by 100 msec MS/MS analyses on the 20 most intense peaks seen by MS. The MS/MS data file generated was analysed using the Mascot algorithm (Matrix Science). Peptides identified above 95% confidence level and with at least 2 hits were analysed.

### Western blot analysis for Ku 70 and Ku 80 expression

Protein extracts were separated by SDS-polyacrylamide gel electrophoresis using a 10% gel and transferred to PVDF. Antibodies used were anti-Ku 70 (Abcam, Cambridge, UK) at a dilution of 1 in 5000 and anti-Ku 80 (Abcam, Cambridge, UK) at a dilution of 1 in 3000. Proteins were visualised using the ECL plus kit (GE Healthcare, Buckinghamshire, UK).

### Aptamer binding selectivity to patient tissue samples

Ethical approval and informed consent was obtained by Cambridgeshire 1 research ethics committee for the use of human tissues to the Brain Tumour North West (BTNW) research bank (Approval reference no. 09/H0304/88). A confirmation letter was issued to the BTNW research bank stating the approval of participants written consent form for the collection of tumour and non-cancerous brain tissues for future use. The current study was approved by the Brain Tumour North West research committee for the use of serial tissue sections from 56 total patients including grade I glioma (n = 7), grade II glioma (n = 14), grade III glioma (n = 10), grade IV glioblastoma (n = 12), and non-cancerous brain (n = 13).

### Dewaxing and antigen retrieval of paraffin embedded sections

Tissue samples were formalin-fixed and serially sectioned (4 μm), then deparaffinised with two changes of histoclear, 15 minutes each and rehydrated through graded ethanol washes (100%, 90% and 70%), 5 minutes each. Deparaffinised tissue slides were rinsed in distilled water. Antigen retrieval involved immersing the slides in 0.01 M citrate buffer at 97°C for 20 minutes. The sections were allowed to stand at room temperature for 30 minutes to cool and then were rinsed in 0.1 M PBS, pH 7.4 twice for 2 minutes each.

### Aptamer labelling and imaging

To mask endogenous biotin, sections were treated with biotin-blocking solution (Vector laboratories, UK) for 30 minutes and then washed three times with 0.1 M PBS, pH 7.4. To eliminate non-specific DNA binding, sections were blocked with 100 μg/ ml salmon sperm DNA for 30 minutes at room temperature. The tissue slides were incubated with 100 nM biotin labelled DNA aptamers for 60 minutes at room temperature and then washed three times with 0.1 M PBS, pH 7.4 for 5 minutes followed by incubation with Vectastain ABC reagent for 30 minutes at room temperature. After three washes with 0.1 M PBS, pH 7.4, the tissues sections were treated with 200 μl of DAB peroxidase substrate solution (Vector laboratories, UK) for 5 minutes at room temperature for colour development. Counterstaining of the cell nuclei in tissue sections was performed with Mayer’s haematoxylin solution for 5 minutes. The slides were then rinsed with distilled water and dehydrated through increasing series of ethanol washes (70%, 90% and 100% two times for 2 minutes each). The sections were placed into xylene for 5 minutes and mounted in DPX mounting medium and were then imaged and captured using bright field microscopy using Nikon microscope equipped with digital eclipse camera system (Nikon, UK).

### Analysis of aptamer binding selectivity and intensity in tissue sections

A United Kingdom National External Quality Assessment Service (UK NEQAS) approved scoring system was applied to analyse and count the cells stained with each DNA aptamer in the tissue sections (Table 2) [36]. The stained tissue sections were blindly scored without knowing the grade of the tissue. A statistical test using one-way ANOVA followed by Bonferroni post hoc test was applied to compare the overall mean binding scores for each aptamer on all glioma versus the non-cancerous tissues. Comparison of binding selectivity between the control (non-cancerous) group and tumour groups (grade I, grade II, grade III and grade IV) were further analysed by Fishers’ exact test (Graph Pad Prism software, La Jolla, USA) using a 2 x 2 contingency table. A value of *p* < 0.05 was considered to indicate a statistically significant difference of each aptamer between the control and tumour group.

**Table 2:** Scoring system.

Score for staining intensity	Score for proportion intensity
0 = no staining	0 = no staining
1 = weak staining	1 = <1% cell staining
2 = intermediate/ moderate staining	2 = 1–10% cell staining
3 = intense/ strong staining	3 = 11–33% cell staining
	4 = 34–66% cell staining
	5 = 67–100% cell staining

## Results

### Binding analysis of SA43 and SA44 aptamers on live U87MG glioma cells

**Fig 1:**
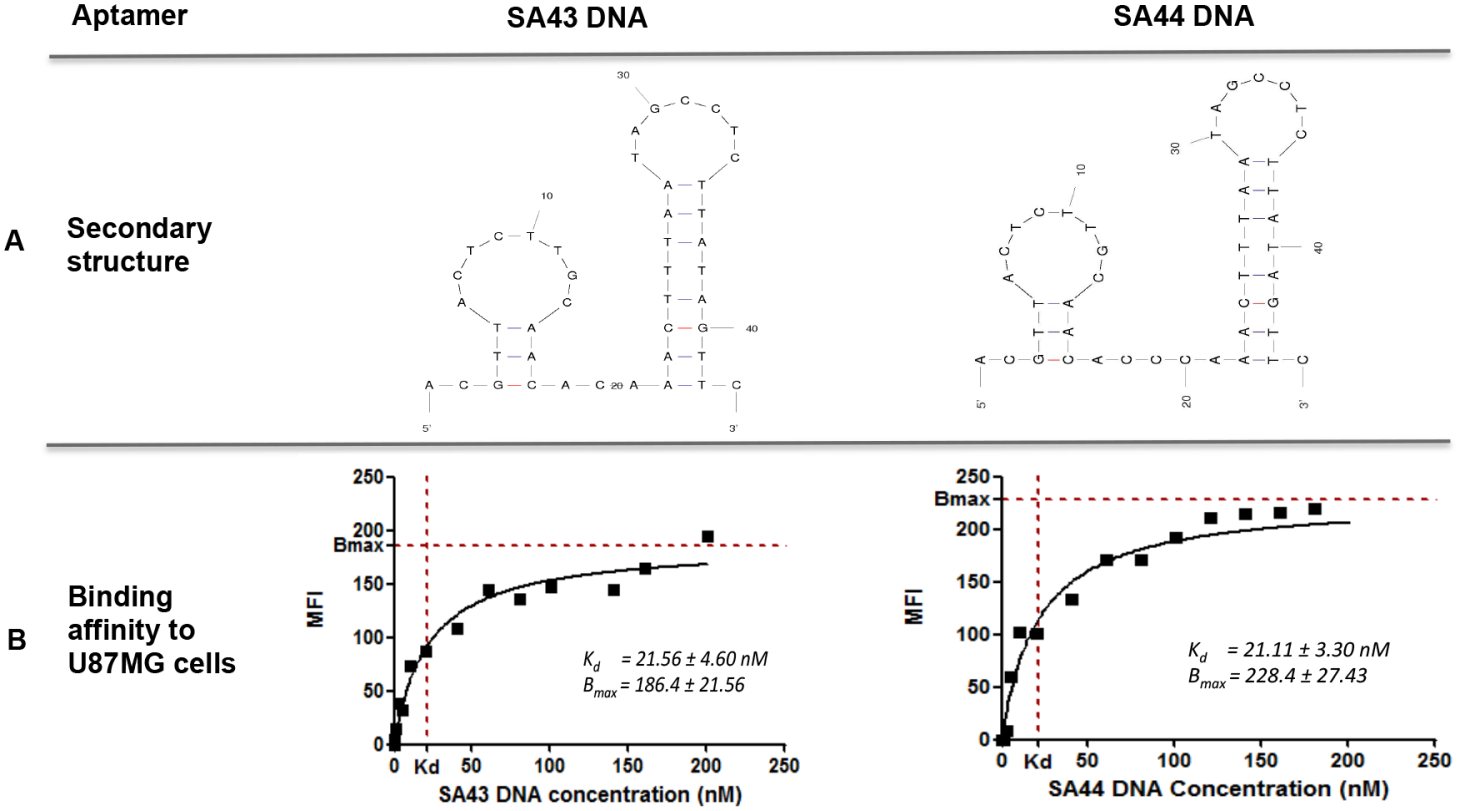
Predicted secondary structures and binding affinity to U87MG cells of aptamers SA43 and SA44 DNA. **A** represents similar secondary structures of SA43 and SA44 aptamers predicted by the M-fold software (Zuker, 2003). Dashes between nucleotides indicate Watson-Crick base pairing. **B** represents binding affinity of aptamers SA43 and SA44 to live U87MG cells analysed by flow cytometry. The mean fluorescence intensity (MFI) of the cells was plotted against varying concentrations of the Cy3 labelled aptamers (0.5–200 nM) and analysed using non-linear regression analysis.

Aptamers are typically between 40–200 nucleotides in length but only specific nucleotides in the loop and bulge region are likely to be responsible for binding. Analysis of SA43 and SA44 sequences using M-fold software showed similar two-dimensional (2D) secondary structures, including two defined conserved stem-loop structures which are likely to be responsible for binding to the target molecule (Fig 1A). After analysing the secondary structure of the aptamers, the binding affinity to the target U87MG cells was assessed by non-linear regression analysis using aptamer concentrations ranging from 5 nM to 200 nM. The binding assay showed that the SA43 and SA44 aptamers bound with high affinity to U87MG target cells in the nanomolar range (SA43, 21.56 ± 4.60 nM and SA44, 21.11 ± 3.30 nM) (Fig 1B).

### Selective uptake of aptamers by various cell types

Confocal microscopy was used to confirm the concentration dependent uptake and to determine the localisation of the aptamers in U87MG cells. Live U87MG cells were incubated with Cy3- labelled aptamers with increasing concentrations ranging from 5 nM to 100 nM for 90 min at 37°C and then analysed using confocal microscopy. The results from Z stacks imaging indicated that the uptake was concentration dependent with lower uptake on lower concentrations and higher uptake at higher concentrations. Negligible uptake was observed at lowest concentration 5 nM. Minimal binding was detected at 20 nM in the cytoplasm, however, both aptamers accumulated in the cytoplasm with increasing aptamer concentration above 20 nM. Maximum Cy3 fluorescence signal was observed from 80 nM in the cytoplasm for aptamers SA43 and SA44 DNA (S1 and S2 Figs). The 100 nM aptamer concentration showed the best contrast between unstained nucleus and stained cell cytoplasm, suggesting that the aptamers were mainly localised in the cell cytoplasm. The binding assay (Fig 1B) also aided in determining the optimum concentration of each shortened Cy3-labelled aptamer for binding live U87MG cells. This was accomplished using flow cytometry to measure the aptamer concentration at which K_d_ values started reaching saturation point. The mean fluorescence intensity (MFI) values of the Cy3 labelled aptamers bound to the live U87MG cells increased with an increasing aptamer concentration until reaching the plateau phase at 100 nM after 90 min incubation, hence this concentration was chosen for further analysis.

**Fig 2:**
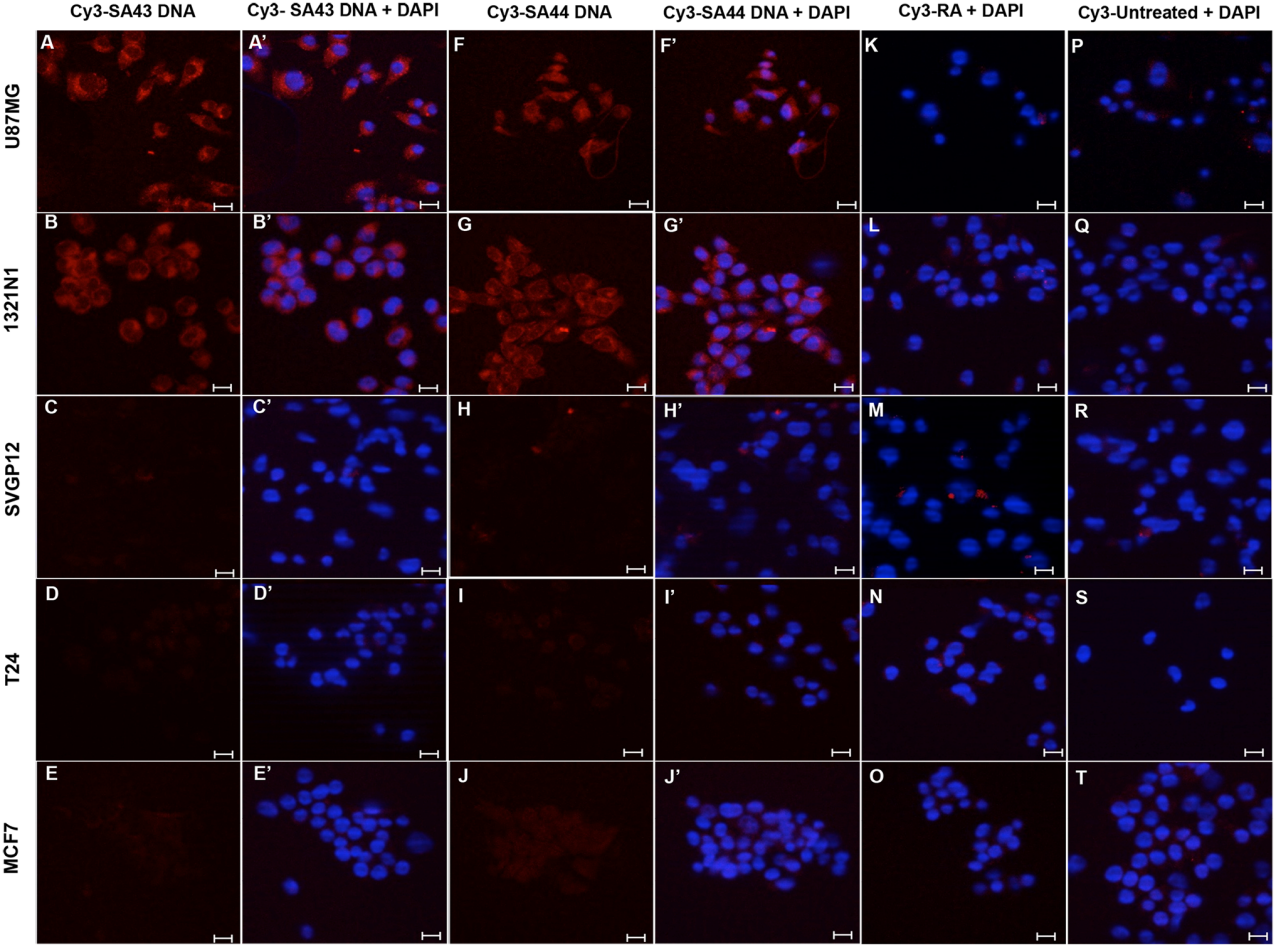
Selective uptake of SA43 and SA44 DNA on various cell lines using confocal microscopy. Cy3 labelled SA43, SA44 (red) and random aptamer (RA) were incubated separately at a concentration of 100 nM on live U87MG, 1321N1, SVGp12, T24 and MCF-7 cells for 90 minutes at 37°C and fixed using 4% PFA. The nuclei were counterstained with DAPI (blue). Cells with random aptamer and media alone were used as control. Z-stacks were acquired under 40x magnifications at an excitation of 543 nm and emission of 560 nm under 1047-camera resolution. Data are representative of three independent experiments with single plane images of middle section of Z- axis. The Cy3 fluorescence signal of the SA44 aptamer on each cell line were compared to the random aptamer and respective untreated controls. **A**(**A’**) and **B**(**B’**) represent Cy3 and merged DAPI image showing high uptake and cytoplasmic binding of SA43 in U87MG cells and 1321N1 cells respectively. **C**(**C’**), **D**(**D’**), and **E**(**E’**) represent Cy3 and merged DAPI image of SA43 showing negligible uptake in SVGp12, T24, and MCF-7 cells, respectively. **F**(**F’**) and **G**(**G’**) represent Cy3 and merged DAPI image showing high uptake and cytoplasmic binding of SA44 in U87MG cells and 1321N1 cells respectively. **H**(**H’**), **I**(**I’**), and **J**(**J’**) represent Cy3 and merged DAPI image of SA44 showing negligible uptake in SVGp12, T24, and MCF-7 cells, respectively. **K**, **L**, **M**, **N**, and **O** represent merged Cy3 and DAPI image of random aptamer (RA) showing negligible uptake and binding on U87MG, 1321N1, SVGp12, T24, and MCF-7 cells, respectively. **P**, **Q**, **R**, **S**, and **T** represent merged Cy3 and DAPI image of untreated U87MG, 1321N1, SVGp12, T24, and MCF-7 cells, respectively. Bar = 20 μm.

**Fig 3:**
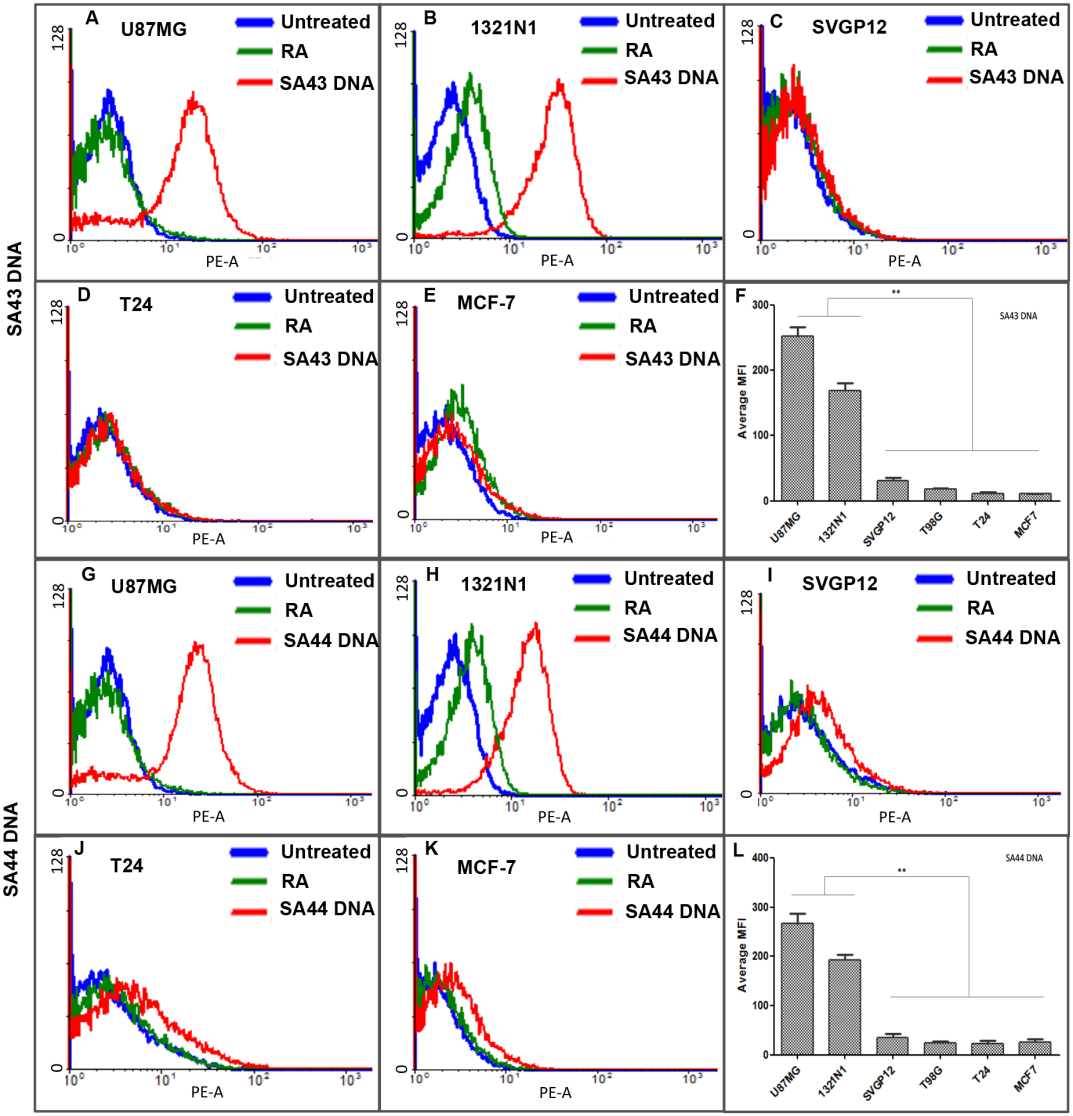
Representative flow cytometry profiles and comparative binding selectivity of the uptake of SA43 and SA44 DNA on various cell lines. Cy3 labelled SA43, SA44 and random aptamer (100 nM) were incubated separately on live U87MG, 1321N1, SVGp12, T24 and MCF-7 cells at 37°C (5% CO_2_) for 90 minutes. Untreated cells were incubated with complete growth medium alone. The blue curve represents the background mean fluorescence intensity (MFI) of the untreated cells (control), and the green and red curve represents the MFI (uptake) of the cells treated with random aptamer and the lead aptamers respectively. **A**, **B**, **C**, **D**, and **E** represent uptake of SA43 on U87MG, 1321N1, SVGp12, T24 and MCF-7 cells respectively. **G**, **H**, **I**, **J** and **K** represent uptake of SA44 on U87MG, 1321N1, SVGp12, T24 and MCF-7 cells respectively. **F** and **L** are bar charts showing comparative binding selectivity of aptamer SA43 and SA44 across various cell types. The background MFI values for the random aptamer treated cells were subtracted. Statistical significance of the differences in MFI values of SA43 and SA44 between U87MG and 1321N1 versus SVGp12, T24, and MCF-7 confirm high binding selectivity of the aptamers for U87MG and 1321N1 cells (** = *p* < 0.01). No statistical difference was observed between U87MG and 1321N1 cells for both the aptamers (*p* > 0.05; n = 3).

The identification of a small set of aptamers SA43 and SA44 DNA which showed high affinity for U87MG glioma cells raised an obvious question of whether these aptamers may bind to other cell types and whether they were able to discriminate cancerous from non-cancerous cells. To this aim, the cell type selectivity was determined by measuring the uptake of each aptamer at the same concentration, 100 nM, on a panel of unrelated cell lines. The uptake and binding selectivity of the aptamers was measured by confocal microscopy and was further confirmed by flow cytometry. Glioma cell lines U87MG and 1321N1 exhibited detectable Cy3-fluorescence (binding) in the cytoplasm for SA43 and SA44 aptamers compared to the respective untreated controls and the random aptamer (Fig 2AA’, 2BB’, 2FF’ and 2GG’). Minimal fluorescence was observed in SVGp12, T24 and MCF-7 cells (Fig 2CC’, 2DD’, 2EE’, 2HH’, 2II’ and 2JJ’). The data showed that SA43 and SA44 were taken up into the cytoplasm and were selective towards glioma cell lines compared to non-cancerous, and non-glioma cell lines. The cell type selectivity and uptake was further confirmed by flow cytometry (Fig 3). A one-way ANOVA followed by post hoc Bonferroni test revealed that SA43 and SA44 showed significant differences in MFI values generated from U87MG and 1321N1 compared to SVGp12, T24 and MCF-7 cells (*p* < 0.01) (Fig 3F and 3L). There was no significant difference observed in MFI values between SVGp12, T24 and MCF-7 cells (*p* > 0.05). This confirmed that DNA aptamers SA43 and SA44 demonstrated significant binding selectivity for U87MG and 1321N1 cells compared to other cell lines.

### Sub-cellular localisation

**Fig 4:**
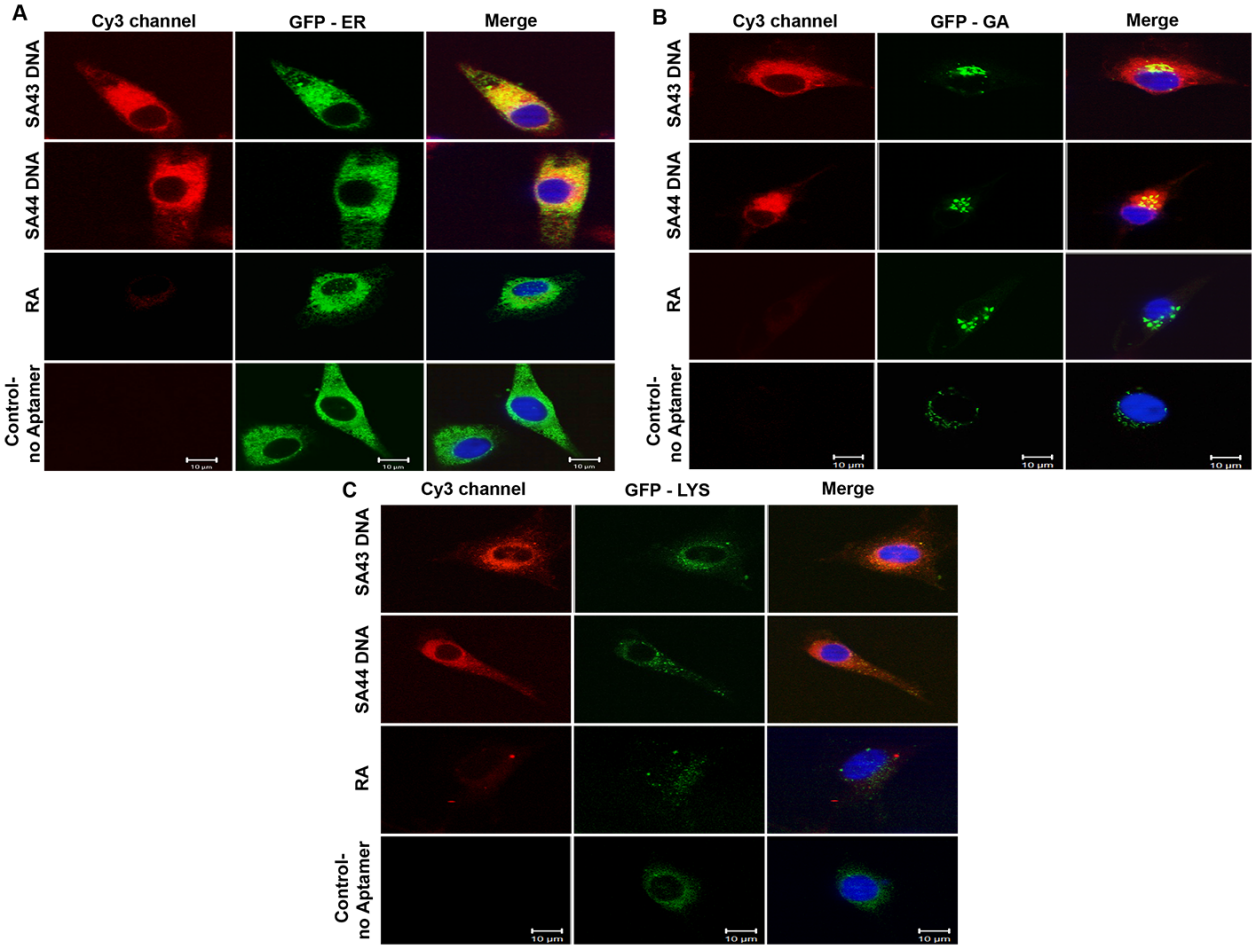
Subcellular localisation of SA43 and SA44 with endoplasmic reticulum (ER), golgi apparatus (GA) and lysosomes. Live U87MG cells were incubated separately with Cy3 labelled SA43, SA44 and RA at a concentration of 100 nM for 24 hours. CellLight ER and GA labelled with GFP, and lysotracker green DND-26 were then incubated to the cells to track the co-localisation of aptamers with ER, GA and lysosomes respectively, and fixed using 4% PFA. The nuclei were counterstained with DAPI (blue). Cells with random aptamer and marker alone were used as control. Bar = 10 μm.

The fluorescence pattern of U87MG cells treated with Cy3 conjugated aptamers included nuclear voids and displayed a reticular pattern of fluorescence not typical of cytosolic localisation. To investigate the sub-cellular localisation further co-staining with organelle-specific fluorescent dyes was used to highlight intracellular organelles; the endoplasmic reticulum (ER), Golgi apparatus (GA) and lysosomes (Fig 4A, 4B and 4C). Both SA43 and SA44 co-localised with ER, Golgi and lysosome markers, confirming its compartmentalisation within the endomembrane system.

### Effect of temperature on aptamer binding to cells

**Fig 5:**
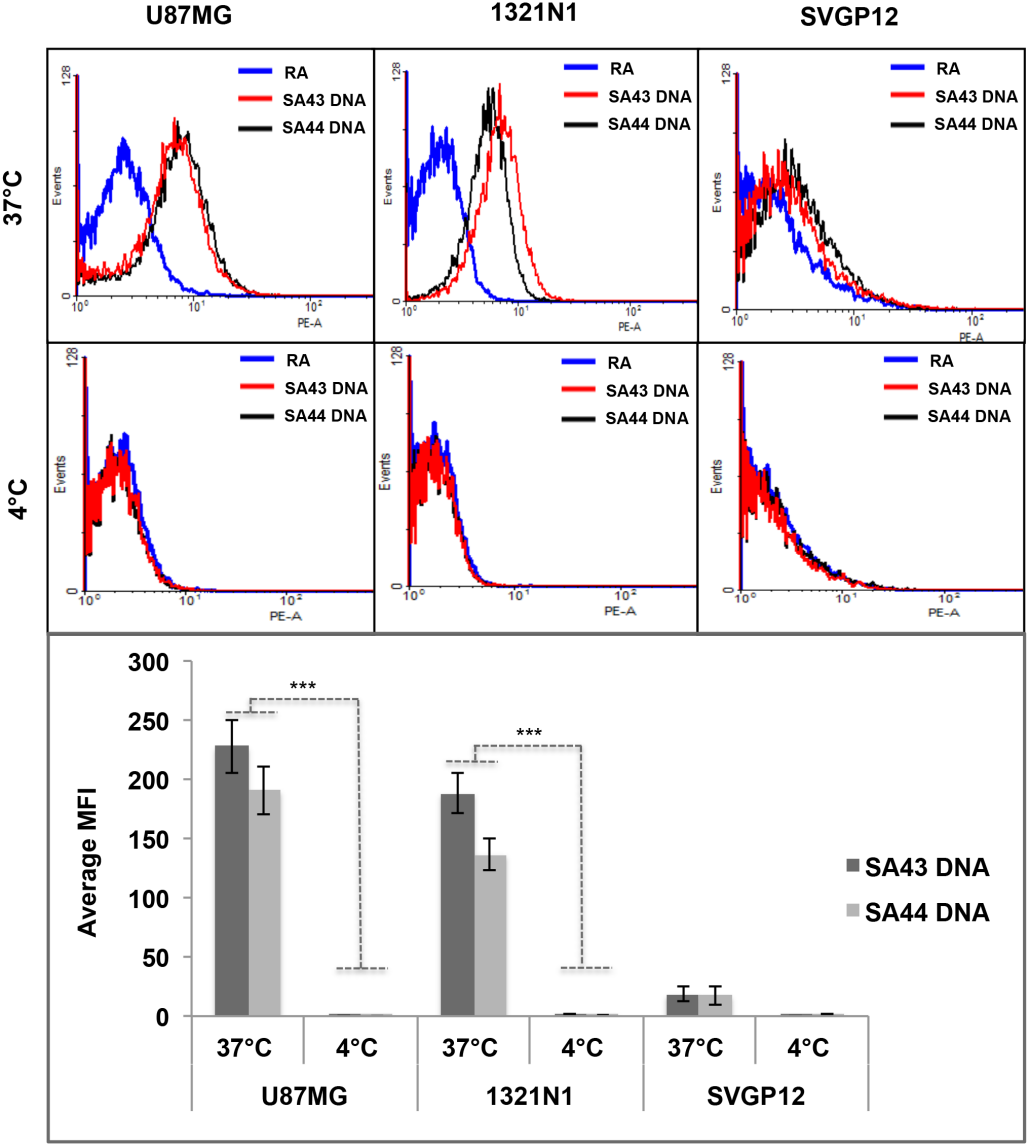
Representative flow cytometry profiles and comparative uptake showing temperature dependent uptake of SA43 and SA44 DNA. Cy3 labelled SA43, SA44 aptamers and random aptamer (RA) (100 nM) were incubated separately with live U87MG, 1321N1 and SVGp12 cells at 37°C (5% CO_2_) or 4°C for 90 minutes. The blue curve represents the background mean fluorescence intensity (MFI) of the random aptamer. The red and black curve represents the MFI (uptake) of the cells treated with SA43 and SA44 aptamer, respectively. **A**, **B** and **C** represent uptake of SA43 and SA44 aptamers on U87MG, 1321N1, and SVGp12 cells, respectively at 37°C. **D**, **E** and **F** represent negligible uptake of SA43 and SA44 aptamers on U87MG, 1321N1 and SVGp12 cells, respectively at 4°C. **G** is a bar chart showing comparative uptake of SA43 and SA44 aptamers. The background MFI values for the random aptamer (control) on corresponding cells were subtracted. Statistical significance of the differences in MFI values of SA43 and SA44 aptamers confirm the high uptake by U87MG and 1321N1 cells at 37°C compared to 4°C by a two way ANOVA test (*** = *p* < 0.001). All experiments were repeated at least three times. Data are mean of three independent samples; bars, SEM.

To investigate whether the selective uptake of aptamers by glioma cells is an active process, the effect of temperature on aptamer binding to cells was determined by flow cytometry. Active and passive transport of any test molecule or drug by cells have been assessed with the commonly used method of parallel incubations at 37°C and 4°C, as active transport proteins are highly temperature dependent [37,38]. Aptamers were added to live cells at either 37°C or 4°C and were incubated for 90 minutes (Fig 5). A two-way ANOVA test revealed that both SA43 and SA44 aptamers showed significantly higher uptake in U87MG and 1321N1 cells at 37°C compared to 4°C (*p* < 0.001) (Fig 5G).

### Effect of aptamers on cell viability

**Fig 6:**
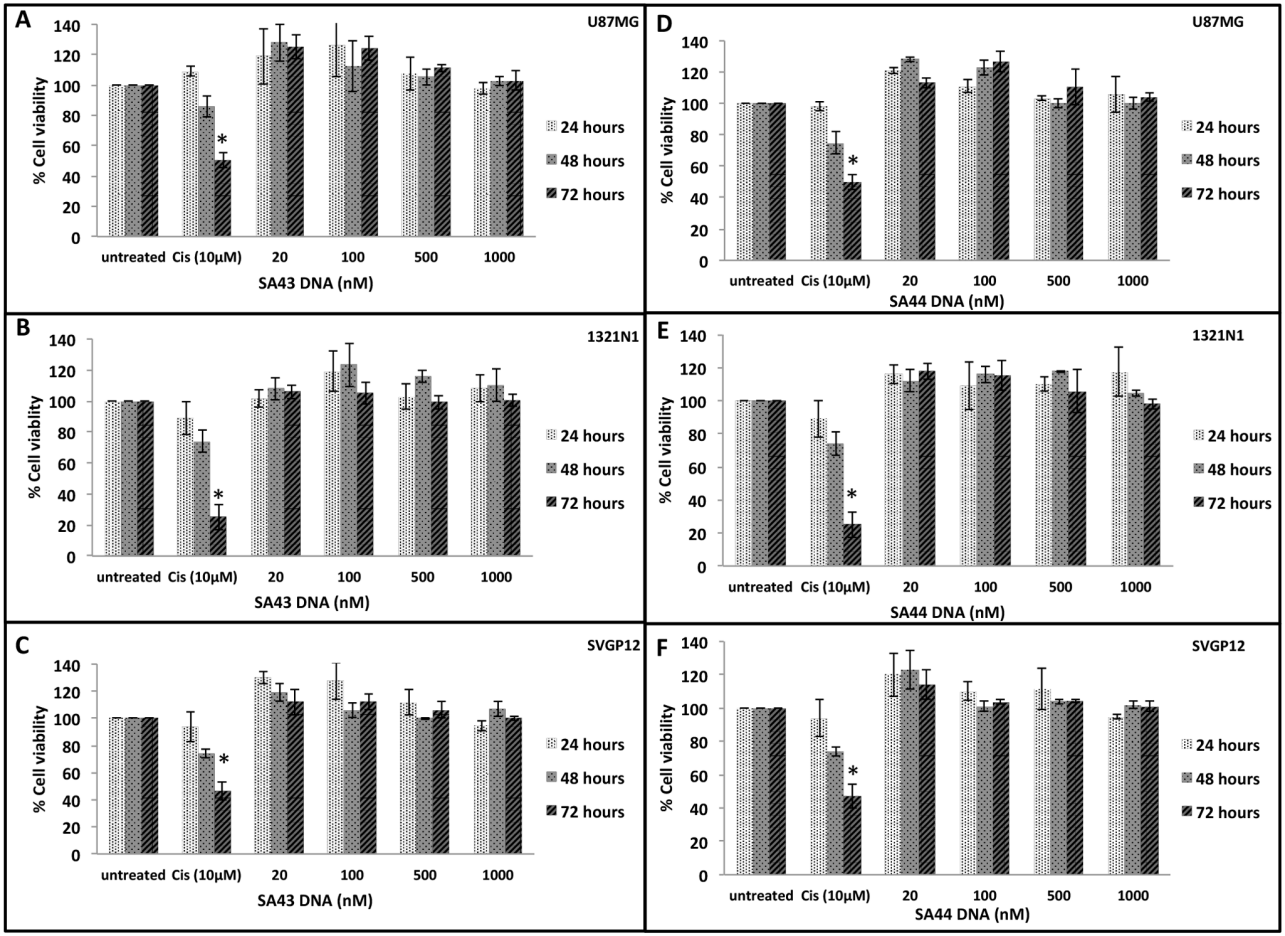
Effect of SA43 and SA44 DNA on cell viability using PrestoBlue assay. Live cells U87MG, 1321N1 and SVGp12 were incubated separately with SA43 and SA44 aptamer at concentrations of 20 nM, 100 nM, 500 nM and 1000 nM at 37°C (5% CO_2_) and assessed for proliferation activity at 24, 48 and 72 hours using the PrestoBlue assay. Cisplatin at 10 μM concentration was used as positive control. A two-way ANOVA test was performed to analyse the difference in the percentage cell viability of the SA43 and SA44 against cisplatin and untreated controls. **A**, **B**, and **C** represent no statistical difference between the percentage cell viability of SA43 and untreated controls for U87MG, 1321N1 and SVGp12 cells, respectively (*p* > 0.05). **D**, **E**, and **F** represent no statistical difference between the percentage cell viability of SA44 DNA and untreated controls for U87MG, 1321N1 and SVGp12 cells, respectively (*p* > 0.05). Cisplatin showed a significant decrease in the percentage cell viability at 72 hours when compared to SA43 and SA44 and untreated control (* *p* < 0.05). All experiments were repeated at least three times. Data are mean of three independent samples. Error bars = Standard deviation.

After establishing the internalisation properties of aptamers, it was important to assess the function of SA43 and SA44 aptamers in terms of their effect on cell viability. This is because aptamers with selective inhibitory potential could be beneficial for therapy if such aptamers can selectively inhibit tumour cell growth [39]. In addition, aptamers with minimal cytotoxicity can be developed for the delivery of therapeutic agents such as small molecule drugs, radioisotopes, toxins, miRNAs and siRNAs into target cells [40–44]. From the study, there was no significant change in viability of cells treated with SA43 or SA44 over a course of 72 hours and up to a concentration of 1000 nM (Fig 6). Microscopic observations also revealed that all cells appeared healthy after aptamer treatments without noticeable morphological changes (data not shown). As expected, cells treated with cisplatin (positive control) showed a significant decrease in cell viability at 72 hours.

### Target identification

**Fig 7:**
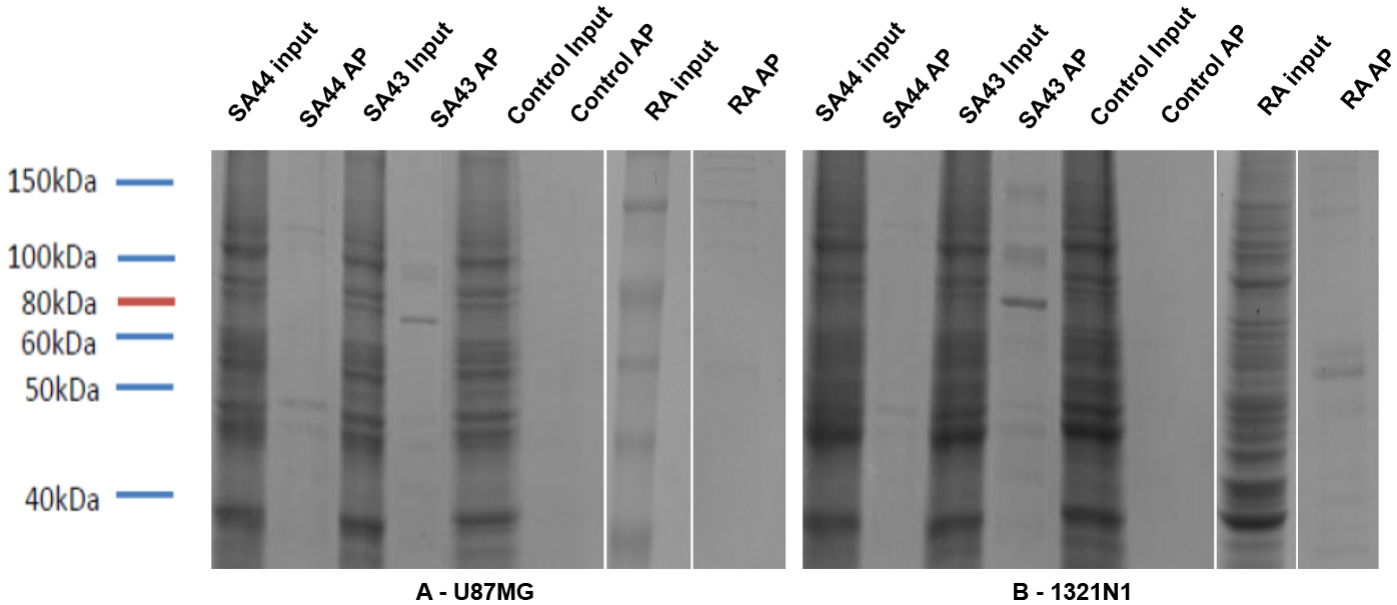
Aptamer precipitation using SA43 and SA44 resulted in the purification of a number of proteins from U87MG and 1321N1. Aptamer precipitation (AP) with either SA43 or SA44 was performed with streptavidin agarose beads with protein extracts prepared from either U87MG (A) or 1321N1 (B). Whole-cell extracts (input) and aptamer precipitated proteins were visualised using comassie blue stain. The control AP contained no aptamer and random aptamer (RA).

**Fig 8:**
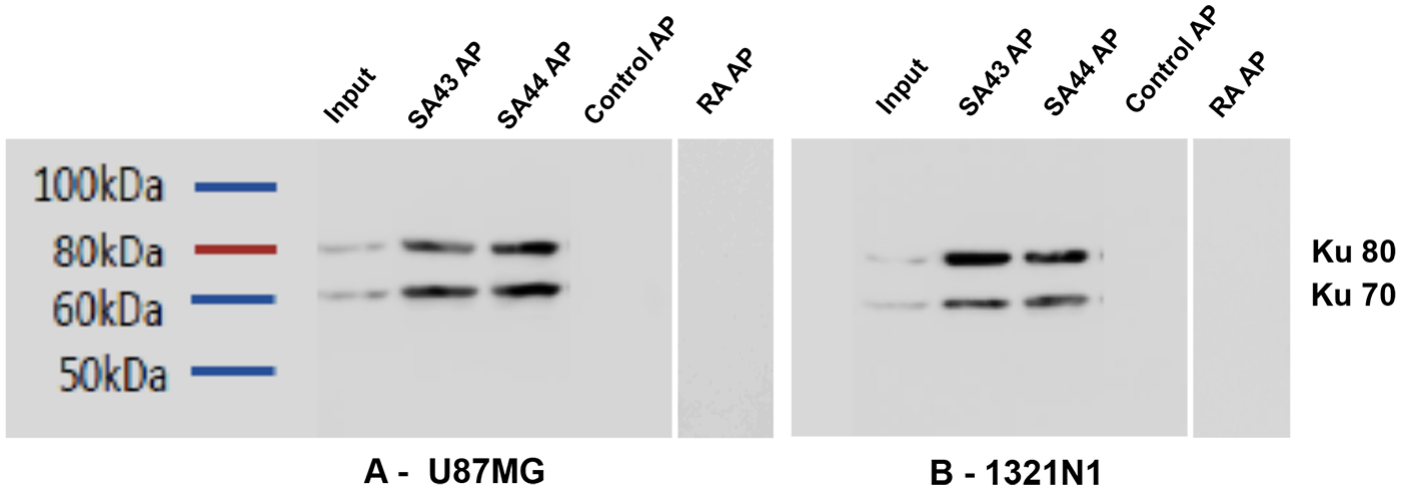
Aptamers, SA43 and SA44, bind to the DNA repair proteins, Ku 70 and Ku 80. Aptamer precipitation (AP) with either SA43 or SA44 was performed with streptavidin agarose beads with protein extracts prepared from either U87MG or 1321N1. Whole-cell extracts (input) and aptamer precipitated proteins were analysed by immunoblotting (WB) using Ku 70 and Ku 80 antibody sequentially on the same blot. The control AP contained no aptamer and random aptamer (RA).

For target identification, the aptamers were used to precipitate proteins from cell lysates. SDS PAGE gels (Fig 7) analysed by LC-MS/MS [45], showed Ku 70 and Ku 80 to be present in significant amount and quantity. Following aptamer precipitation (AP) with SA43 and SA44 on U87MG and 1321N1 cell lysate, the Ku 70 and Ku 80 targets were confirmed by Western blot with Ku 70 and Ku 80 antibodies (Fig 8). Expression in the AP was much higher than the input (cell lysate) indicating specific attachment of the aptamer to the protein with a clear control with random aptamer and no aptamer.

### Binding analysis of DNA aptamers on fixed clinical tissues

**Fig 9:**
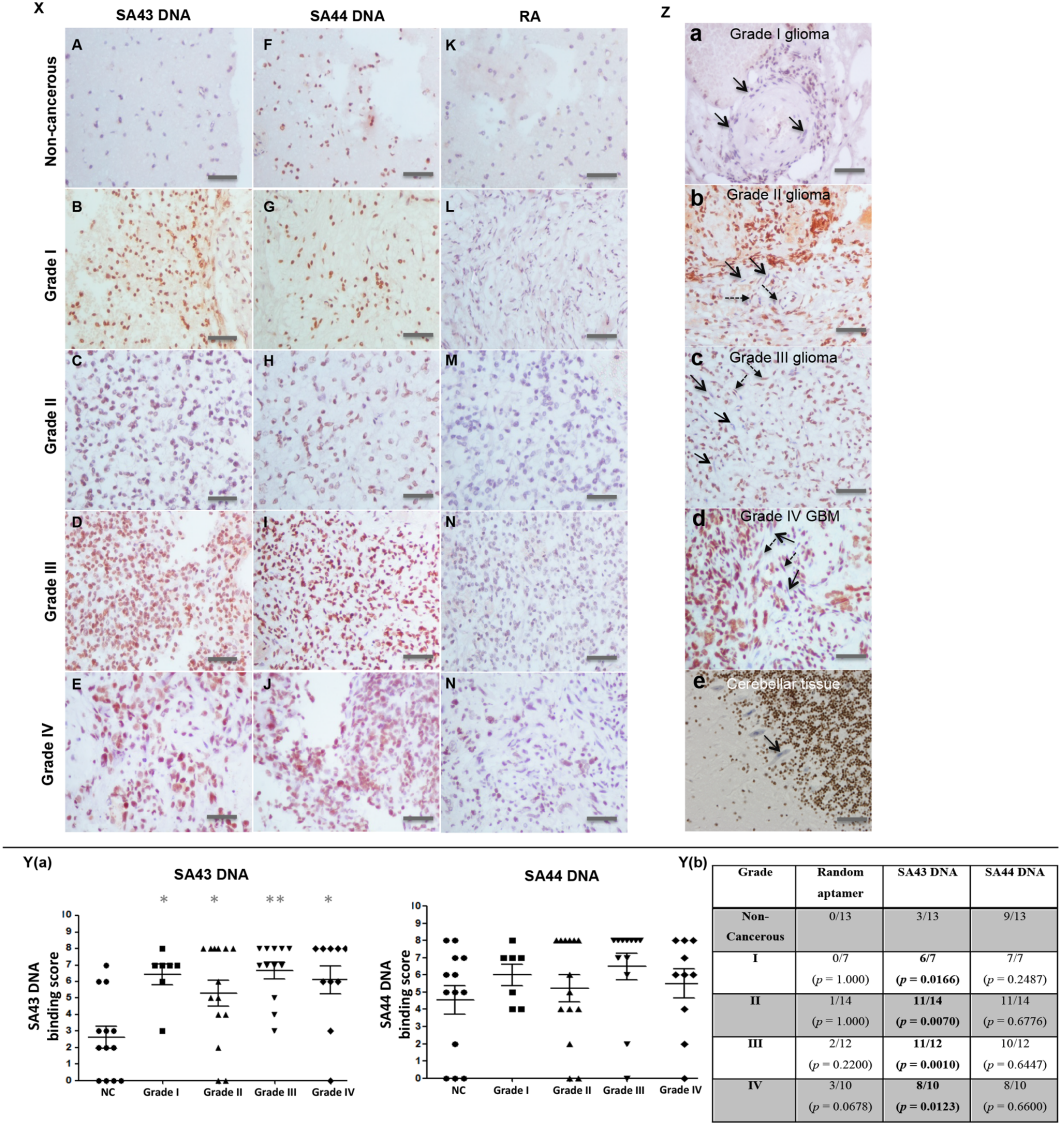
Aptohistochemistry analysis of non-cancerous and different pathological grade glioma patients stained with biotinylated DNA aptamers. **Panel X** Representative images of tissue sections from non-cancerous and different pathological grade glioma patients stained with biotinylated SA43, SA44 and random aptamer (RA). **A-E** SA43 treated; **F-J** SA44 treated; **K-N** RA treated. **Panel Y (a)** Scatter plot showing the distribution of average scores for each aptamer on all patient tissue sections. Using ANOVA and post hoc Bonferroni test, SA43 showed statistical difference in the average binding scores between all pathological grades (I, II, III, IV) of glioma tissues compared to the non-cancerous (NC) tissues (*, *p* < 0.05; **, *p* < 0.01). **Panel Y (b)** Fishers exact test confirmed significant difference in binding selectivity between non-cancerous group and tumour groups with grade I, II, III, and IV treated with SA43 aptamer (*p* < 0.05) (highlighted in bold). SA44 showed no statistical difference in the average binding scores between tumour and NC tissues. **Panel Z** SA43 showing cell type selectivity within the tissue. Endothelial cells are indicated by arrows in grade I glioma (**a**), grade II glioma (**b**), grade III glioma (**c**), and grade IV GB (**d**) and show a mix of positive (dash arrows) and negative (straight arrows) staining with SA43 aptamer. **e** represents negligible binding to Purkinje cells (straight black arrow). All scale bars, 200 μm.

Having determined the selectivity, uptake and target of aptamers in glioma cell lines, the study was extended to clinical tissue sections from different patient samples to determine the effectiveness of the aptamers in recognising and differentiating between different grades of glioma and non-cancerous brain tissues. Fig 9X shows representative images of tissue sections from non-cancerous and different pathological grades of glioma tissues stained with biotinylated DNA aptamers. Staining of tissue sections was graded using a UK NEQAS scoring system. SA44 showed moderate to strong nuclear staining in glioma tissues with an average score ranging from 5.3–6.5, however, most of the non-cancerous tissues also showed strong nuclear staining with the SA44 aptamer with an average score of 4.5. With regards to SA43, there was differential staining on tumour tissues when compared to non-cancerous tissues. The aptamer showed moderate to strong nuclear staining in tumour tissues with an average score ranging from 4–6.6, whereas, in non-cancerous tissues, the average score was only 2.9. The random aptamer showed comparatively negligible binding to the non-cancerous and tumour tissues.

To identify a clinically meaningful cut off point for defining positive binding of aptamers to the cells on tissue sections, the results were examined on a scatter plot (Fig 9Y(a)). Positive aptamer binding was defined if the total aptohistochemical (AHC) score was greater than 3 and negative aptamer binding if the total score was less than or equal to 3. Scores of 2 or 3 did match non-cancerous tissue samples which included patients with less than 1% or 10% cells weakly or moderately stained. Moreover, due to the risk of obtaining false positive results, it was inappropriate to include patients with less than 1% to 10% weakly stained cells with the groups showing more than 10% staining. Comparison between the control (non-cancerous) group and tumour groups (grade I, grade II, grade III and grade IV) were analysed using Fishers’ exact test (Fig 9Y(b)). A significant difference in binding selectivity between non-cancerous group and tumour groups (grades I, II, III and IV) was observed with SA43 aptamer (*p* < 0.05). SA44 and the random aptamer showed no significant difference in binding selectivity between non-cancerous brain tissues and all grades of glioma. This indicated that SA43 could potentially discriminate between all glioma grades and non-cancerous tissues. SA43 showed variable binding to endothelial cells of all of the glioma tissues from all the pathological grades (Fig 9Z(a–d)) but negligible staining to Purkinje cells (Fig 9Z(e)). The overall findings from the study showed that SA43 DNA could be utilised for AHC to potentially discriminate between glioma tissues and non-cancerous brain tissues.

## Discussion

The overall aim of the study was to examine the potential of DNA aptamers as a targeting agent for glioma cells and tissues. Taking into account the available literature, it was hypothesised that aptamers can be applied for cancer diagnosis [31,33,46–48].

Aptamer-target complexes often reveal low dissociation constants that range from nanomolar to picomolar levels [31–33]. The results reported here suggested that the DNA aptamers would be suitable as a molecular probe for recognition and targeting of glioma cells as both aptamers not only showed strong affinity to the target glioma cells with nanomolar dissociation constants, but also an ability to actively internalise rapidly into live tumour cells compared to the non-cancerous and non-glioma cells. The stronger binding affinity of the shortened DNA aptamers to U87MG cells compared to their full length counterparts (GL43 44 ± 4 nM and GL44 38 ± 3 nM) [32] is likely to be attributed to the unique nucleotide sequence and therefore different folding characteristics. In addition, because of the relatively small size compared to the full length aptamers [32], SA43 and SA44 may exhibit superior cell penetration, a property that can help the aptamers to internalise rapidly into the cancer cells [49].

Sub-cellular distribution of aptamers SA43 and SA44 in U87MG cells was assessed by confocal microscopy and revealed reticular staining throughout the cell with a more punctate pattern towards the cell periphery. These patterns were indicative of localisation to organelles of both the endocytic and secretory pathways. To confirm, co-localisation analysis was performed and demonstrated that both aptamers were located in the ER, Golgi apparatus and lysosomes. It is therefore likely that aptamers are being internalised via endocytosis and subsequently passed to the secretory pathway, however further studies will be required to confirm this.

Flow cytometry confirmed quantitatively that SA43 and SA44 aptamers bound and internalised into glioma cells at 37°C when compared to the non-cancerous and non-glioma cells. This is because the cells, after incubation with aptamers, were challenged with protease trypsin to collect the cells for flow cytometry which could potentially degrade all aptamers bound to the cell surface receptors [33,50–52]. Therefore, the signal (MFI) was from the cells that allowed the aptamers to internalise. Even if the aptamers were capable of binding to cell surface at 4°C and not able to internalise, cell trypsinisation for flow cytometry analysis would have caused the loss of extracellular components of membrane proteins, because of which no uptake was observed. This was also supported by a reduction in glioma cellular uptake of the aptamer at 4°C when energy dependent processes, including endocytosis, are reduced. Although it is recognised that passive diffusion is also reduced at 4°C, it is unlikely the aptamer is up taken by a passive process because of the saturable binding observed at the higher aptamer concentrations in the binding affinity assay. Moreover, target identification (discussed in more detail below) supported the hypothesis that aptamer uptake is active and highly selective to glioma cells. Selective internalisation of the aptamer to glioma cells would be of great benefit in designing strategies for targeted therapy of glioma, as it would improve drug accumulation in targeted glioma cells and tissues thus enhancing the targeted therapy.

Ku 70 and Ku 80 were identified as the most abundant target pull down in the samples analysed by mass spectrometry, followed by subsequent confirmation by Western blot using commercially available antibodies. Ku 70 and Ku 80 are ubiquitously expressed DNA repair proteins [53] which are present as a heterodimer in the non-homologous end joining (NHEJ) pathway [54]. Ku 70 and Ku 80 are overexpressed in a number of cancer cells including breast [55], cervix [56], head and neck [57], colorectal [58], prostate [59] and brain [60,61]. Ku 70 and Ku 80 are predominantly localised in the nucleus, however, in a number of cancer cells these proteins re-localise to the cytoplasm and cell membrane [61–64]. This is likely due to the attempt at DNA repair by the cell during cancer progression. The localisation of Ku heterodimer outside of the nucleus in tumours suggests that this protein is serving additional roles besides its main function in DNA repair such as cell adhesion, migration and invasion, however, these roles as yet are poorly understood [65]. Ku 70 and Ku 80 overexpressed in breast cancer [55] and bladder cancer tissues [64], raised an obvious question for the negligible binding of aptamers on live MCF-7 and T24 cells. Biopsy tissue represents a heterogeneous cell population and cell lines are not truly representative of the parent tumour because they may mutate in culture. In addition, expression of Ku 70 and Ku 80 is highly dependent on the genotype of the cancer such as *BRCA1*, *HER2* and *RAD51* status [55]. All these implications can result in false positive results and therefore, binding studies in breast cancer and bladder cancer tissues will confirm whether the aptamers are truly selective towards glioma or whether the aptamers can be used to distinguish other types of cancer from their non-malignant tissue counterparts. The Ku heterodimer is a DNA binding protein and as such the binding of SA43 and SA44 could be non-selective. This is, however, unlikely as a random DNA aptamer did not aptoprecipitate with the Ku heterodimer. Further, the random aptamer did not show binding to glioma cell lines or patient tissue which would have been expected if Ku 70/80 non-selectively binds to DNA aptamers. In addition, for the patient tissue samples, salmon sperm DNA was used to block non-specific binding which would have reduced the ability of a non-selective interaction between the aptamers and DNA binding proteins. As Ku 70/80 is ubiquitously expressed in all cells, if it non-selectively bound to SA43 we would have expected staining of all cells within the tissue samples. As only a sub population was detected, which increased in both intensity and proportion with severity of the glioma, this supports that notion that SA43 selectively binds to Ku 70/80. The overexpression of Ku 70/80 in cancer tissues serves as a useful biomarker for diagnosis and targeted therapy, thus the generation of aptamers that are capable of binding to such targets to distinguish tumour cells from non-cancerous cells in heterogeneous tissue samples has immense clinical potential.

Few aptamers with cell-internalising properties such as E07 and pegaptanib have been shown to inhibit the cell growth [66,67], whereas, numerous studies based on aptamers have provided a proof of concept that aptamers with minimal cell inhibitory properties can mediate cell type-specific delivery through conjugation with toxins, chemotherapeutic drugs, imaging agents and siRNAs [68–71]. The DNA aptamers studied here showed no growth inhibitory effect on cells suggesting they possess negligible cell inhibitory effect at the concentrations tested and are therefore ideal candidates for delivery of therapeutic payloads to the targeted site. This could reduce any harmful side effects, as only cancerous cells would be targeted rather than all proliferating cells.

Having confirmed the selectivity and non-toxicity of both SA43 and SA44 aptamers to glioma cell lines, we sought to elucidate the application of aptamers in AHC. An established scoring system was used as it was found to be highly reproducible, correlated with established biochemical assays and provided equally significant predictive and prognostic information regarding patient samples [36,72]. The optimal cut off point in the study was a total AHC score of greater than 3, which considered more than 10% cells positively stained with the aptamers. This was in agreement with many clinical and commercial laboratories choosing 10% or even 20% positive tumour cells as the cut off value for defining oestrogen receptor (ER), progesterone receptors (PR), and HER2 positivity in cancer tissues [36,72,73]. Using this scoring system, the study showed differential binding of the SA43 aptamer on tumour tissues compared to the non-cancerous tissues. SA44 reflected SA43 in binding to glioma tissues; however, it also showed binding to most of the non-cancerous tissues. SA44 differed in the presence of nucleotide bases ‘CC’ at 19^th^ and 20^th^ positions compared to SA43, however, the absence of nucleotides did not alter the secondary structure. Whether the binding of SA44 to the non-cancerous tissues is due to the presence of nucleotides bases ‘CC’ still remains an unsolved question. Structural studies with aptamer-target interactions have demonstrated diversity in tertiary structures of aptamers associated with folding upon binding to the target [74]. If this was the case for SA44 and SA43 aptamers, the deletion of the two cytosines from the SA44 sequence might cause a different 3-dimensional (3D) conformation to be adopted more preferable for targeting tumour cells. On the other hand, SA43 aptamer demonstrated an ability to detect the glioma cells in brain tissues, whereas SA44 did not differentiate between glioma and non-cancerous tissues despite identifying the same target(s); Ku 70/80. This difference in discrimination capabilities could be due to aptamers binding to different epitopes of the Ku 70/80 protein or, alternatively due to the level of sensitivity in the AHC; SA44 may be more sensitive than SA43 and is therefore able to detect lower (i.e. normal) levels of Ku 70/80.

As glioma progressed to a higher grade, the aptamers showed higher AHC score which could be contributed to higher localisation of target cells or higher expression of target proteins to be present in higher grade compared to the lower grade. Considering that Ku 70/80 is also expressed predominantly in the nucleus of non-cancerous cells and tissues, the absence of binding to non-cancerous cells and tissues with SA43 DNA aptamer demonstrated aptamer selectivity to detect the target in glioma and hence could be utilised in histological glioma diagnosis. Moreover, given the ease of synthesis *in vitro*, smaller size, non-immunogenicity, no batch to batch variation, reduced cost, potentially increased stability and shelf life of aptamers compared to antibodies along with reduced time to perform the AHC technique when compared to antibody based staining, SA43 DNA will potentially be advantageous in future clinical settings by overcoming the limitations of antibodies. The study therefore focussed on SA43 DNA aptamer, as it undoubtedly showed similar binding characteristics on both live cells and fixed clinical tissues.

A number of aptamers have been reported to bind targets on endothelial cells of brain tumour with a view to distinguishing them from the endothelial cells in non-cancerous brain [75,76]. The study here however showed binding of SA43 aptamer to some endothelial cells. The fact that not all endothelial cells were bound by SA43 aptamer could be regarded as a hindrance if this aptamer was to be used for targeted drug delivery across the blood brain barrier (BBB). The molecular weight of the SA43 DNA aptamer (14 kDa) may also preclude the aptamer from crossing the BBB (cut off is 0.4–0.6 kDa) through transcellular transport unless specific transporters are present. Our parallel studies, however, have shown that SA43 is able to cross an *in vitro* human 3-dimensional BBB as determined by a temporary reduction in the trans-endothelial electrical resistance (TEER) and an apparent permeability of 4.32 ± 3.90 x 10^−9^ cm/ min (unpublished data). It is unknown at this stage whether the aptamer is able to cross through the BBB via transcellular transport, but the variable binding to endothelial cells seen with SA43 suggests that the aptamer is able to bind to endothelial specific ligands within the cell. The active binding selectivity for glioma tumour cells and ability to cross an artificial BBB indicates that SA43 may hold a powerful promise in improving targeting glioma cells for either therapeutic or diagnostic purposes. The next stage is to test the ability of fluorescently tagged aptamers in animal model systems to dynamically visualise and target the tumour tissues *in vivo*.

Overall, our data demonstrates that the SA43 aptamer is selective for glioma when compared to the non-cancerous tissue. In summary, the results provided a proof-of-concept for the use of DNA aptamers to target tumour cells in both research and clinical settings. The assays undertaken have confirmed the internalisation potential, cellular localisation and target of the selected aptamers. The selective binding capability of aptamers studied, suggested that the interaction of the aptamer and target largely depends on the nature of the aptamer sequence. The key consequence of the internalising property of the aptamer SA43 is the ability to accumulate inside the tumour cells, thus this aptamer is an ideal candidate for targeted drug delivery. The selective binding capability of aptamer SA43 and the sensitivity to glioma tissues will hold promise for histochemical diagnosis of glioma. Moreover, the small size of the aptamer, negligible inhibition of cell proliferation, and some binding to endothelial cells will potentially allow the aptamer to cross the BBB. Successful targeted delivery, however, must also satisfy the need for aptamers to have chemical properties that allow long circulation in the blood, maintain biocompatibility, and exhibit control release. The smaller molecular mass for most of the aptamers (5–15 kDa) including SA43 (14 kDa) could be susceptible to renal filtration even if they are resistant to nuclease degradation. To overcome such difficulties, pyrimidine modifications at the 2’-fluorine position or chemical modifications with PEG have been used to enhance the bioavailability and pharmacokinetic properties [77]. This will allow further testing of potential aptamer-drug conjugates for their enhanced efficacy in targeting tumour cells *in vivo*. In addition, much work remains to be done before these conjugated materials can be used in clinical practice such as understanding the transport of aptamers to the cancer locale, the interactions between aptamers and cancer cells, and the subcellular density. The study utilised paraffin-embedded formalin fixed tissue samples for determining the potential of aptamers in histological diagnosis of glioma. Use of such fixed tissues however, may have a possibility of altering the composition or structure of epitopes during the tissue processing methods. Use of unfixed frozen tumour tissues over fixed waxed tissues can minimize tissue processing and preserve the natural presentation of epitopes. Treating aptamers on frozen tissues will further assist to acquire clearer conclusions and support the current findings on aptamer targeting to tumour tissues. Importantly, these efforts promise to expedite the development of the aptamer- based approaches for histological diagnostics and also delivering therapeutic drugs to the cytoplasm of the target cells and possibly a more rapid translation for therapeutics to humans.

## Supporting Information

S1 Fig Concentration dependent uptake of SA43 DNA aptamer. (TIF)

S2 Fig Concentration dependent uptake of SA44 DNA aptamer. (TIF)
