# Supplementary figures and images for: Selective Targeting to Glioma with Nucleic Acid Aptamers
Source: PLoS One. 2015 Aug 7;10(8):e0134957. doi: 10.1371/journal.pone.0134957 (PMC4529171; doi:10.1371/journal.pone.0134957)

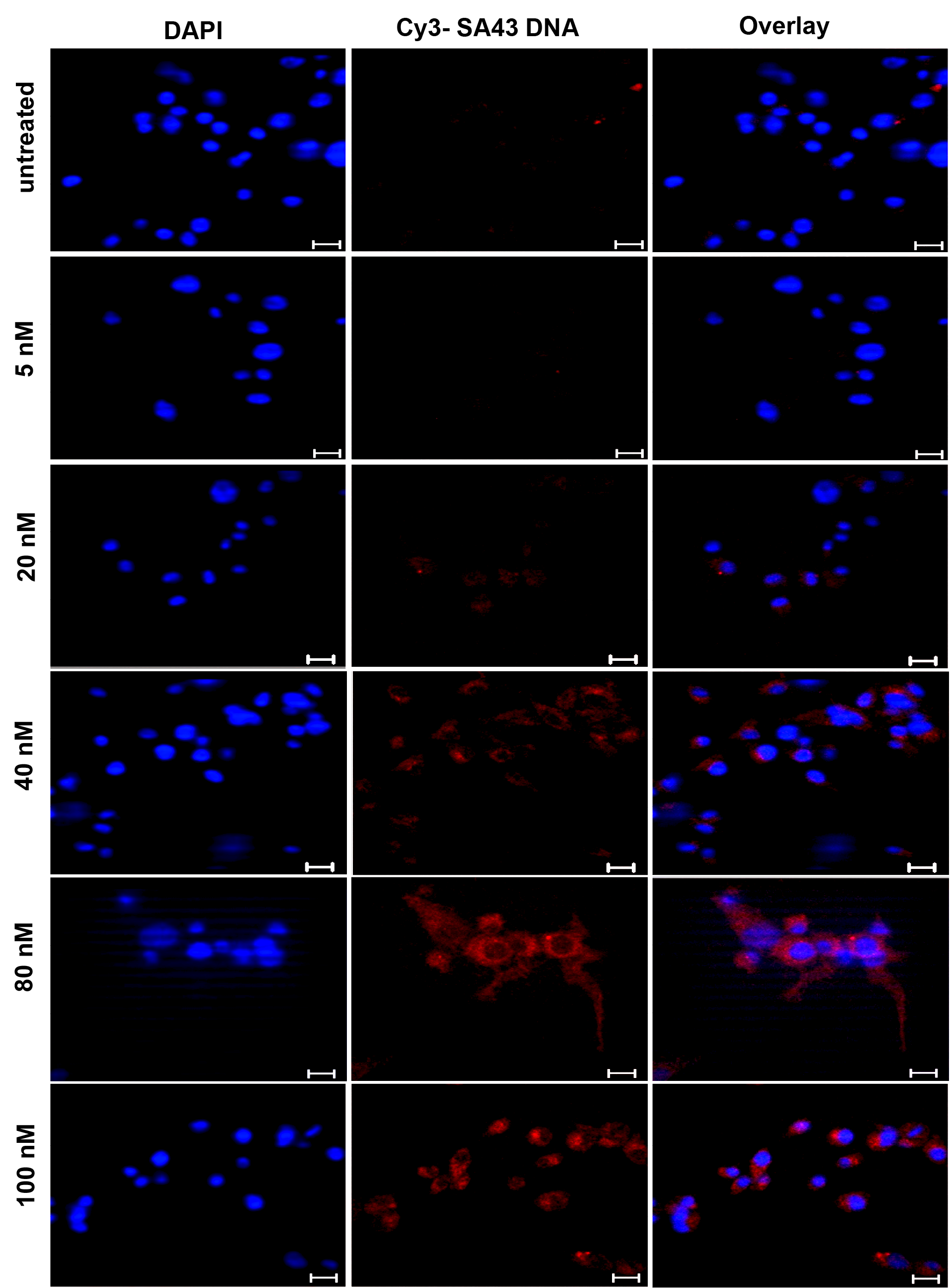

Supplement: S1 Fig — (TIF) [file pone.0134957.s001.tif]

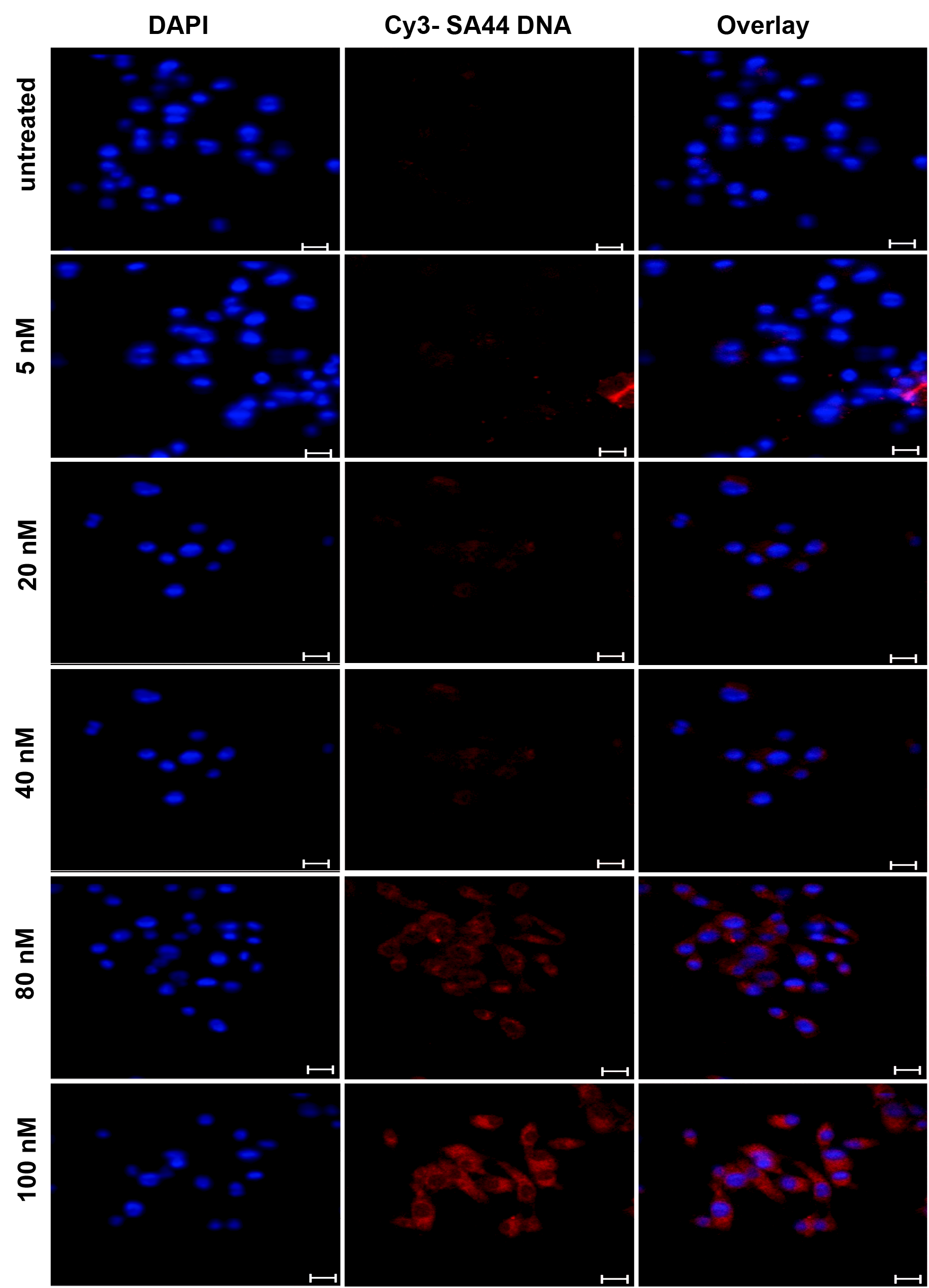

Supplement: S2 Fig — (TIF) [file pone.0134957.s002.tif]
